# Reduction of autofluorescence in whole adult worms of *Schistosoma japonicum* for immunofluorescence assay

**DOI:** 10.1186/s13071-021-05027-3

**Published:** 2021-10-14

**Authors:** Qunfeng Wu, Zheng Feng, Wei Hu

**Affiliations:** 1 State Key Laboratory of Genetic Engineering, Ministry of Education Key Laboratory of Contemporary Anthropology, Collaborative Innovation Center for Genetics and Development, School of Life Sciences, Fudan University, Shanghai, 200438 People’s Republic of China; 2 National Institute of Parasitic Diseases, Chinese Center for Disease Control and Prevention, Key Laboratory of Parasite and Vector Biology of the Chinese Ministry of Health, WHO Collaborating Center for Tropical Diseases, Joint Research Laboratory of Genetics and Ecology On Parasite-Host Interaction, Chinese Center for Disease Control and Prevention & Fudan University, Shanghai, 200025 People’s Republic of China

**Keywords:** *Schistosoma japonicum*, Reducing autofluorescence, Immunofluorescence, Sudan black B, Copper sulfate

## Abstract

Immunofluorescence assay is one of methods to understand the spatial biology by visualizing localization of biomolecules in cells and tissues. Autofluorescence, as a common phenomenon in organisms, is a background signal interfering the immunolocalization assay of schistosome biomolecules, and may lead to misinterpretation of the biomolecular function. However, applicable method for reducing the autofluorescence in *Schistosoma* remains unclear. In order to find a suitable method for reducing autofluorescence of schistosomes, different chemical reagents, such as Sudan black B (SBB), trypan blue (TB), copper sulfate (CuSO_4_), Tris-glycine (Gly), and ammonia/ethanol (AE), at different concentrations and treatment time were tested, and SBB and CuSO_4_ were verified for the effect of blocking autofluorescence in immunofluorescence to localize the target with anti-*Sj*CRT antibody. By comparing the autofluorescence characteristics of different conditions, it was found that SBB, TB and CuSO_4_ had a certain degree of reducing autofluorescence effect, and the best effect in females was using 50 mM CuSO_4_ for 6 h and in males was 0.5% SBB for 6 h. Furthermore, we have applied the optimized conditions to the immunofluorescence of *Sj*CRT protein, and the results revealed that the immunofluorescence signal of *Sj*CRT was clearly visible without autofluorescence interference. We present an effective method to reduce autofluorescence in male and female worm of *Schistosoma japonicum* for immunofluorescence assay, which could be helpful to better understand biomolecular functions. Our method provides an idea for immunofluorescence assay in other flukes with autofluoresence.

**Supplementary Information:**

The online version contains supplementary material available at 10.1186/s13071-021-05027-3.

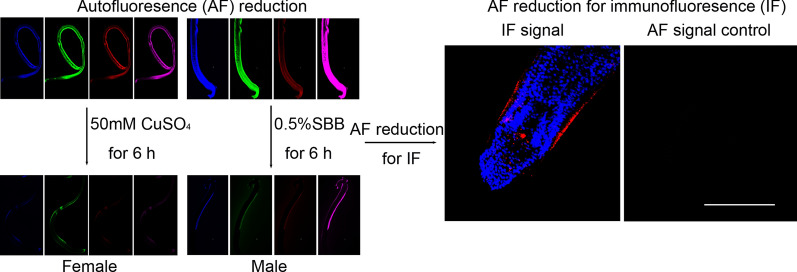

Schistosomiasis is a neglected tropical disease caused by trematodes of the genus *Schistosoma*, imposing great threat to the health of people and animals, as well as impacting economic development [1]. In a host of biological studies, immunofluorescence assay (IFA) has been widely applied to visualize the spatial location of target molecules of interest in a great variety of different types of tissue and cell preparations [2, 3]. The IFA is also an essential tool to identify and localize schistosome molecules of interest in understanding the worm developmental strategy and parasite-host molecular interplay [4–6]. The assay capability is achieved by using fluorophore-labelled antibody to directly or indirectly react with a target antigen in biological samples. However, this technique is disadvantaged by unwanted fluorescence either due to autofluorescence (AF) from the target tissue or the fluorescent background caused by non-specific binding of the fluorescent label. AF interference is one of the shortcomings of IFA particularly when using confocal laser scanning microscopy, which requires minimal tissue AF and reduced non-specific fluorescence background. AF, either intrinsic or induced by fixation processing, may either mask specific fluorescent signals or be mistaken for fluorescent labels [7].

Biological AF, emitting from endogenous fluorophores, is an intrinsic property of cells and tissues, commonly seen in mammalian cells and tissues [8, 9], rodents [10], nematodes [11] and *Schistosoma* [12]. It is also noted that AF property of specific tissue constituents may be of diagnostic value or indicative of cell viability. In schistosomes, the AF of eggs was used to detect eggs in diseased tissues [13]. AF of the vitelline gland in female schistosome emits mainly from vitelline cells, which could be used to separate and enrich vitelline cells [14] or applied for vitelline gland localization [15]. However, AF is often a noise signal in IFA. Therefore, AF has been a significant concern in IFA.

Various histochemical techniques for blocking AF have been evolving. Sudan black B (SBB), trypan blue (TB), copper sulfate (CuSO_4_), Tris-glycine, ammonia/ethanol (AE) have been tried to control the AF [16–18]. The efficacy of chemical reagents in reducing AF differs with the sample type. It was reported that CuSO_4_ was used to quench AF within the vitellarium of *Schistosoma mansoni* [19], but little is known of applicable reagents for reducing the AF in *S. japonicum*.

The present study characterized the AF of male and female *S. japonicum*, and tested five chemical reagents for assessing the efficacy of reducing the AF in IFA.

Parasites and animals were prepared. Female Kunming mice (20–25 g) (Shanghai Animal Center, Chinese Academy of Sciences, China) were infected with 80 ± 5 *S. japonicum* cercariae (provided by the National Institue of Parasitic Diseases, China CDC). Adult worms were harvested by perfusion with ice-cold 0.9% NaCl solution containing heparin (10 U/mL) (Sangon Bioengineering Technical Services, China) at 28 days post-infection. Male and female worms were gently separated, and fixed with 4% paraformaldehyde (Sangon Bioengineering Technical Services, China) for 2 h at room temperature and then kept overnight at 4 °C.

After fixation, the worms were treated with 1% SDS (in PBS) for 20 min. A blocking solution (2% goat serum, 1% skimmed milk powder, 0.1% cold fish skin gelatin, 0.1% Triton-X 100, 0.05% Tween 20, 0.05% NaN3 in PBS) was applied at 4 °C overnight. Worms were washed three times with PBS.

To examine the AF of *S.japonicum*, the fixed male and female worms were mounted on the slide with 80% glycerol (Sinopharm Chemical Reagent, China) and viewed with confocal laser scanning microscope (CLSM) (Nikon A1R, Nikon Instruments, Japan). The fluorescence signals were aligned to four fluorescence channels with the following filter setting of excitation and emission wave length: DAPI, 405–450 nm; EGFP, 488–515 nm; mCherry, 561–610 nm; and AF647, 640–665 nm (Table 1).

**Table 1:** Fluorescence image acquisition using CLSM

Acquisition channel	Fluorophore used	Excitation (nm)	Emission (nm)	Image display color
DAPI	DAPI	405	450	Blue
EGFP	NA	488	515	Green
Cy3	Cy3	532	590	Red
mCherry	NA	561	610	Red
AF647	NA	640	665	Purple

*DAPI* 4′,6-diamidino-2-phenylindole, *Cy3* Cyanine3, *AF647* Alexa Fluor 647, *NA* not applicable

To ascertain effective reagents to control the AF arising from schistosomes, we tested five chemical reagents (Sigma-Aldrich), including CuSO_4_, SBB, TB, Tris-glycine (Gly), ammonia/ethanol at different concentrations and for different treatment time-length. The whole worms were immersed in copper sulfate at 0.5, 5 or 50 mM in 50 mM ammonia acetate for 1.5, 3 or 6 h; in 0.01%, 0.1% or 0.5% SBB in 70% ethanol for 1, 2 or 6 h; with 0.05% TB for 1 or 2 h; with 0.1 M Gly in TBS (pH 7.4) for 2 h; immersed in 0.25% ammonia in 70% ethanol for 2 h. All procedures were performed at room temperature. To remove the excess of testing regents, the worms were washed six times for 20 min each with 0.02% Tween 20 in PBS (PBST). Then the worms were placed on slide, mount the slide with 80% glycerol and viewed with CLSM.

To verify the reactivity of the anti-*Sj*CRT antibodies with crude antigens of *S. japonicum* worms, Western blotting was performed. The worm protein was extracted with 10–20 worms in 1 ml PBS by sonication on ice and then centrifuged for 10 min at 13,000 g, 4 °C. The Western blotting was performed as previously described [20]. The protein extracts (50 μg protein) of adult female and male worms were resolved by 12% SDS-PAGE and electrotransferred onto polyvinylidene fluoride membrane. The membrane was incubated with blocking solution (PBS, pH 8.0, 0.05% Tween 20, 5% skimmed milk) at 4 °C overnight. The membrane was washed three times with PBST (PBS with 0.1% Tween 20), and rabbit anti-*Sj*CRT (*S. japonicum* calreticulin) IgG (Shanghai YouKe Biotechnology, China) diluted at 1: 2000 in PBST was applied for 6 h at room temperature. The blot was then incubated in a blocking solution containing HRP-conjugated goat anti-rabbit IgG (BBI, Shanghai, China) at a dilution of 1: 8000 for 2 h at room temperature. After washing with PBST, the membrane was developed with NcmECL Ultra solution (NCM Biotech, Suzhou, China), and imaged using Tanon 5200 (Tanon, Shanghai, China). The skimmed milk powder, Tween 20 and Triton-X 100 were from Sangon Bioengineering Technical Services, China; goat serum from Zhejiang Tianhang Biotechnology, China; all other reagents were produced from Sigma-Aldrich.

The immunofluorescence assay (IFA) was performed as described in previous report [15]. After fixation, blocking of non-specific antigens, and treatment with the optimal conditions to control AF, the worms were incubated with rabbit anti-*Sj*CRT IgG (1:300–500) in blocking solution at 4 °C for 3 days, followed by washing three times for 2 h each with PBST. For fluorescence staining, the worms were incubated with Cy3-labeled goat anti-rabbit IgG (1:500) in blocking solution at 4 °C for 3 days, and at the end of 2nd day the DAPI staining solution (1:50) was added to the incubation solution. After washing three times for 2 h each with PBST, the worms were placed on slide, mount the slide with 80% glycerol and viewed with confocal laser scanning microscope (Nikon A1R, Nikon Instruments Inc., Japan). In the immunofluorescence assay, the DAPI and Cy3 fluorescent dyes were used for labelling nuclear and *Sj*CRT protein respectively [21, 22], the fluorescence signals were detected in DAPI and Cy3 channels respectively. The EGFP detection channel was used for monitoring green AF and evaluating the reducing AF effect. The fluorescence images of whole-mount worms were examined using CLSM. The DAPI, Cy3 and EGFP channel of microscopy conditions for fluorescence imaging were showed in Table 1. The DAPI was purchased from Boster Biological Technology, China. The Cy3-labeled goat anti-rabbit IgG was from Beyotime Biotechnology, China.

After preparative processing, the worms were mounted with 80% glycerol and placed under a confocal laser microscope for observation. It was observed that both female and male worms had AF in four different channels (DAPI, EGFP, mCherry and AF647). In female worm, AF was seen in the tegument, vitelline gland, ovary, eggs, as well as the worm head (Fig. 1a). However, no blue AF was observed in the vitelline gland, ovary and eggs (Fig. 1a DAPI column). AF in male schistosomes was mainly distributed in the intestine, tegument and gynecophorc canal (Fig. 1b). In male, the fluorescence intensity at the intestine is relatively stronger. In male and female worms, the strongest intensity of AF was seen under the EGFP channel compared to others (Fig. 1).

**Fig. 1:**
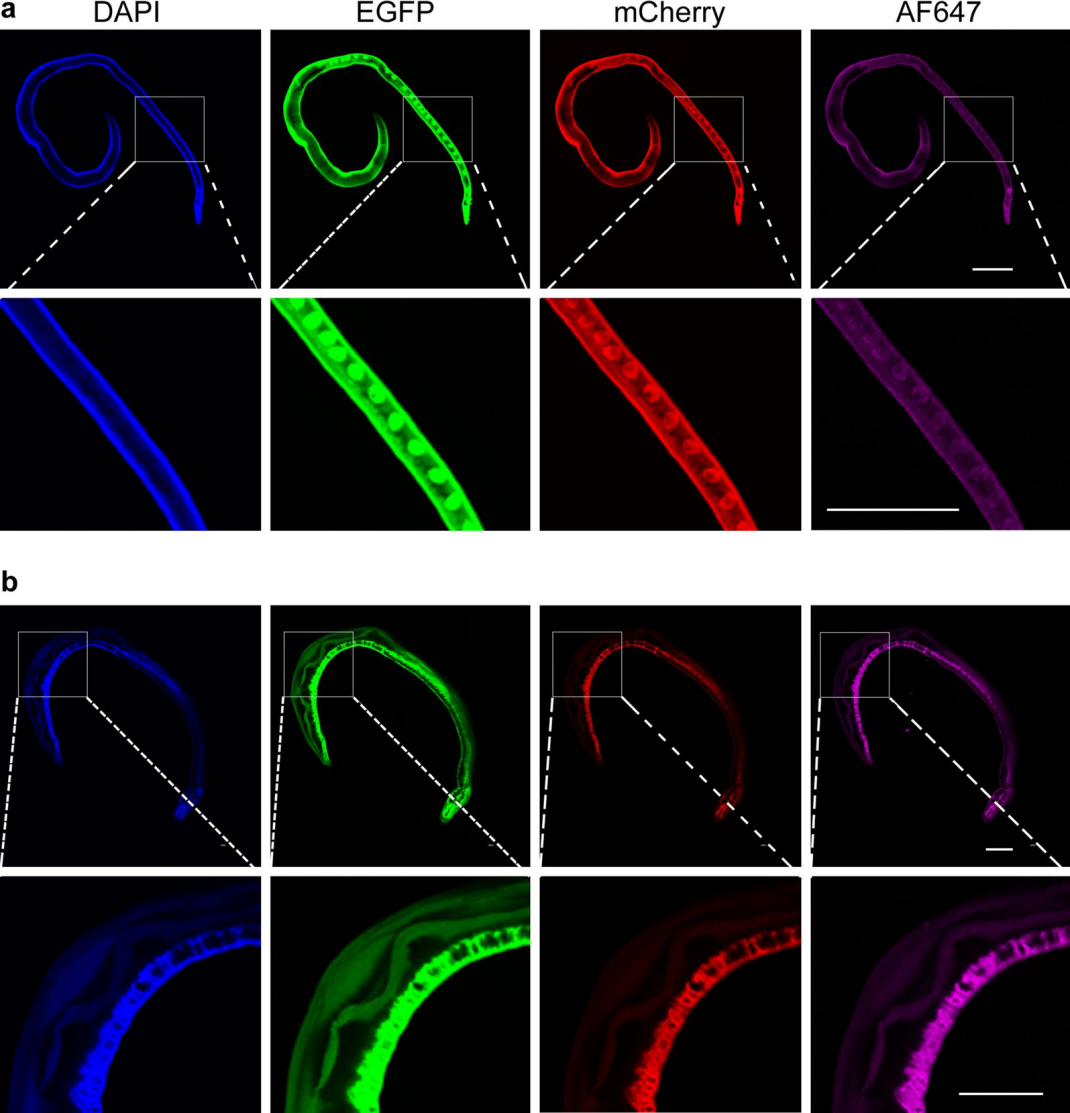
Characteristics of autofluorescence in female and male of *Schistosoma japonicum*. **a** Visualization of autofluorescence in whole female worms under confocal microscopy. **b** Visualization of autofluorescence in whole male worms under confocal microscopy. The imaging conditions for the four channels (DAPI, EGFP, mCherry and AF647) are summarized in Table 1. Scale-bar: 500 μm

Five different chemical reagents were used to treat male and female worms for reduction of AF, and it was found that reduction effect varied with different reagents, among which the effect of Tris-glycine (Gly) and Ammonia/ethanol (AE) was unobvious (Additional file 1: Figure S1), while CuSO_4_, SBB, and TB had some effect (Fig. 2). It was noted that the effect of blocking AF also differed with the worm gender and the time of chemical exposure. After treatment with CuSO_4_, the AF in both male and female worms was reduced, however the reduction was more significant on female worms (Fig. 2b and c). Treated with 5 mM CuSO_4_ for 1.5 h, the AF in females was attenuated in all four tested channels (Fig. 2b), and the treatment for 3 h led to the AF undetectable in mCherry and AF647 channels (Fig. 2c). There was no significant reduction AF in the four channels in males treated with 5 mM of CuSO_4_ for 1.5 h (Fig. 2b), whereas the reduction effect was seen in mCherry and AF647 channels after 3 h of treatment (Fig. 2c).

**Fig. 2:**
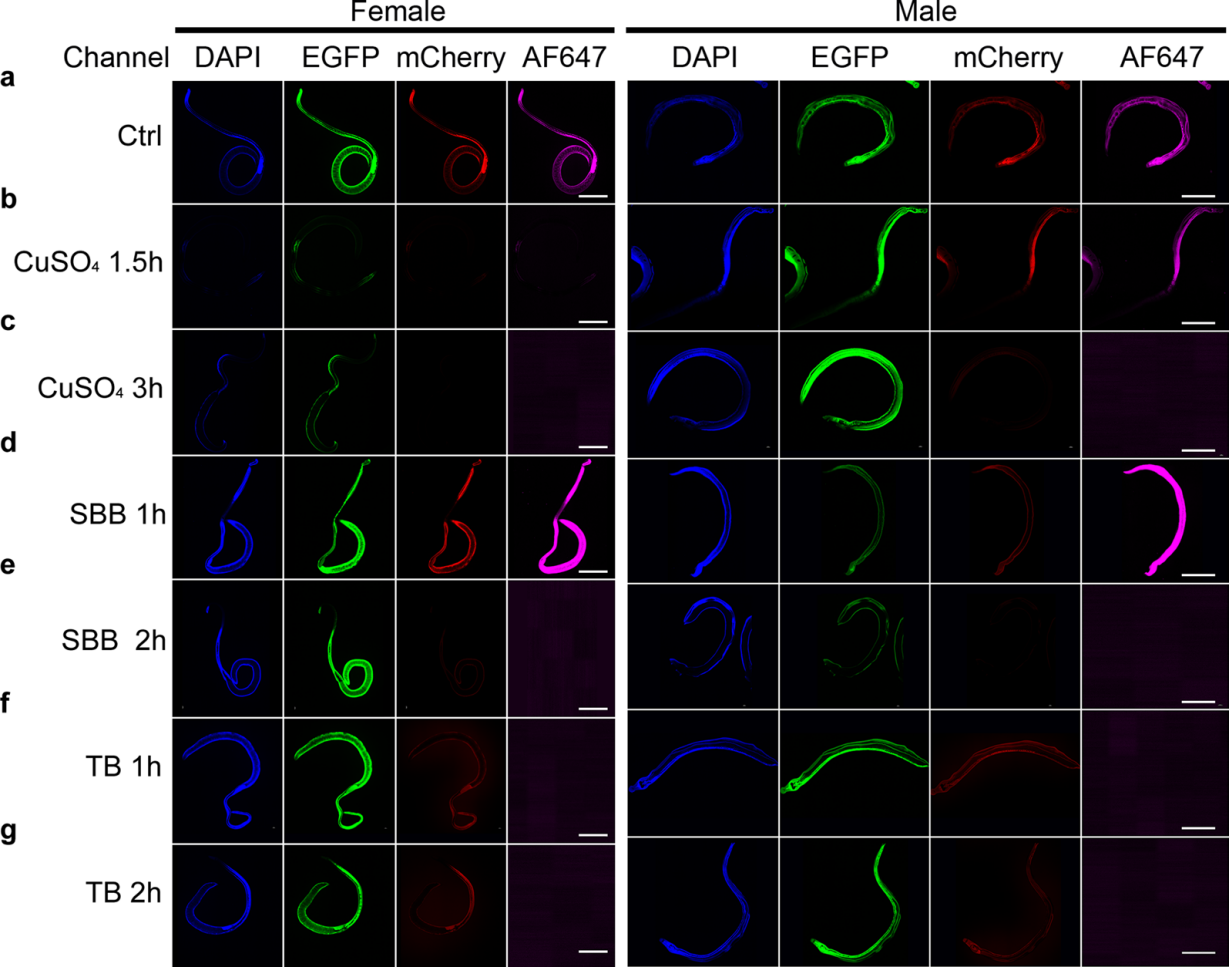
Photographs of *Schistosoma japonicum* treated and untreated with chemical regents. The autofluorescence of *Schistosoma japonicum* (**a**). The effect of 5 mM copper sulfate (CuSO_4_) incubated 1.5 h (**b**) and 3 h (**c**) on autofluorescence. The effect of 0.1% Sudan black B (SBB) incubated 1 h (**d**) and 2 h (**e**) on autofluorescence. The effect of 0.05% trypan blue (TB) incubated 1 h (**f**) and 2 h (**g**) on autofluorescence. Scale-bar: 1000 μm

SBB exhibited some effect in controlling AF in both males and females, particularly in males (Fig. 2d and e). By treatment with 0.1% SBB for 2 h, AF in females was reduced in mCherry channel, while blocked in AF647 channel (Fig. 2e); but treatment with 0.1% SBB for 1 h did not exert perceptible AF reduction in females (Fig. 2d). Interestingly, incubation with 0.1% SBB for 1 h significantly attenuated AF in male worms in EGFP and mCherry channels (Fig. 2d); the AF intensity in males in DAPI and EGFP channels was significantly reduced after treatment for 2 h, while blocked mCherry and AF647 channels (Fig. 2e). TB showed certain reductive effect on AF in male and female worms, but only seen in AF647 channel (Fig. 2f and g).

It was demonstrated that CuSO_4_ presented better reduction effect on AF in female worms and SBB in male worms, and longer time exposure to reagents would enhance the effect of reducing AF. Different concentrations of CuSO_4_ and SBB were used to treat worms and the results are shown in Fig. 3. Comparing with the control, all the defined concentrations of CuSO_4_ and SBB had the AF reduction effect. The reduction effects of AF of female worms were increased with the increase of CuSO_4_ concentration, of which the AF detected in EGFP channel was more difficult to remove than other channels. The effect of AF in male worms was not significantly different between 0.01% SBB and 0.1% SBB, but the effect of 0.5% SBB treatment was significantly better. Among all the chemical solutions, 50 mM CuSO_4_ showed a best AF reduction effect on female worms (Fig. 3a) while 0.5% SBB had a best AF reduction effect on male worms (Fig. 3b).

**Fig. 3:**
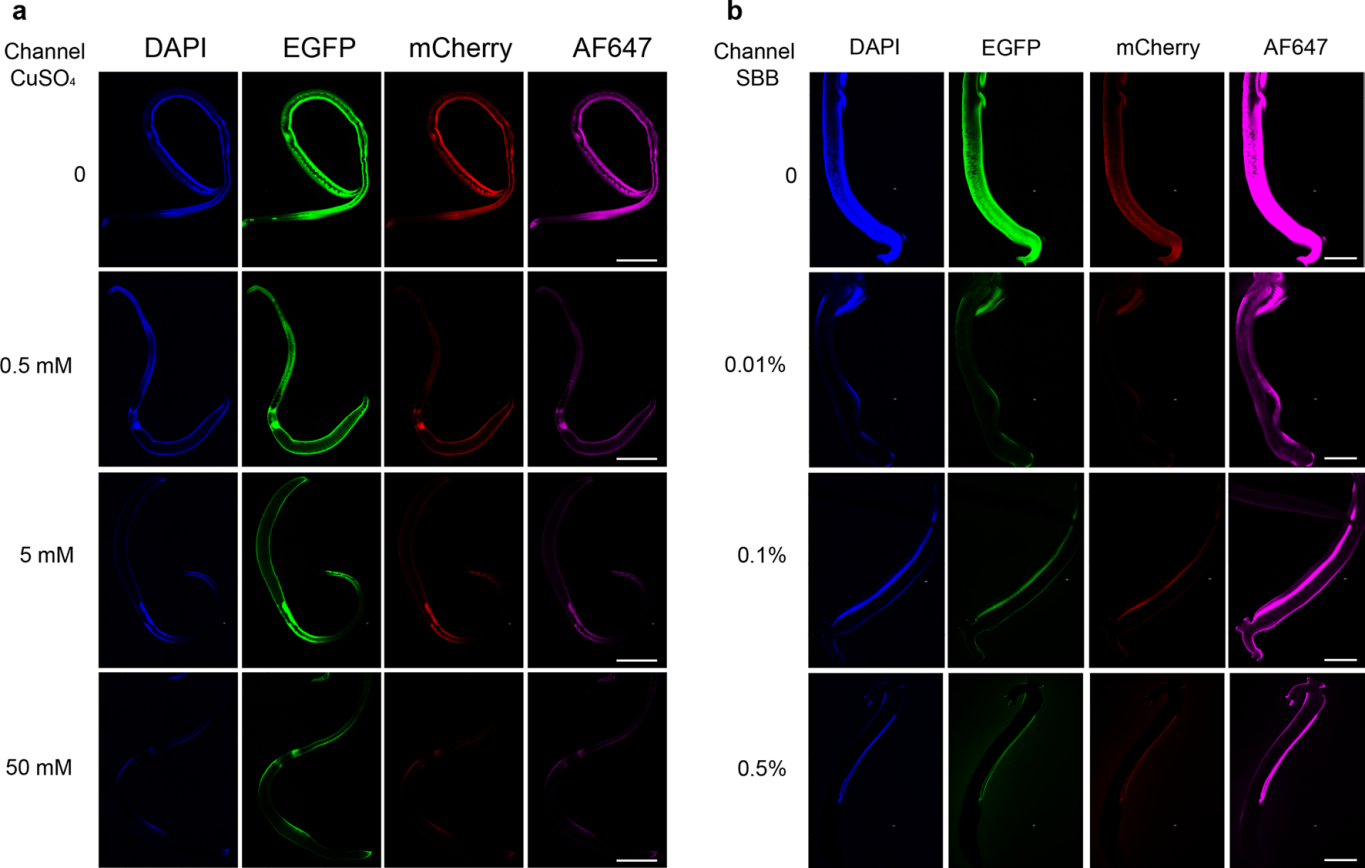
Optimization of AF reduction conditions. **a** Autofluorescence of female worms treated with different copper sulfate (CuSO_4_) concentrations. **b** Autofluorescence of male worms treated with different concentrations of Sudan black B (SBB). Scale-bar: 500 μm

Western-blotting (WB) was used to verify the reactivity of the anti-*Sj*CRT antibodies with crude antigens of male and female worms. The WB results showed a single band in the crude antigen lanes of both male and female worms, with molecular weights around 55 kDa (Additional file 2: Figure S2). The band of the female protein lane was less intense than the male protein lane (Additional file 2: Figure S2).

In order to verify the effect of AF reduction conditions applied in immunofluorescence experiments, we performed immunofluorescence localization assay on *Sj*CRT. In this assay, the fluorescent dye Cy3-labeled goat anti-rabbit IgG was used to localize *Sj*CRT; the nuclear was stained by DAPI. Furthermore, we not only set up a normal rabbit serum control, but also monitored the AF using EGFP channels unoccupied by any fluorescent dye. By the treatment of AF reduction in IFA, no significant AF signal was seen both in females and males under the EGFP channel (Fig. 4a and b, Additional file 3: Figure S3). And in the normal rabbit serum control, non-specific immunofluorescence signal was not detected in females except weak non-specific immunofluorescence signal in males under Cy3 channel (Additional file 3: Figure S3). The nuclear staining and *Sj*CRT IF staining signals were scattered granular both in females and males (Fig. 4), and the weak non-specific immunofluorescence signal did not interfere with the signal of *Sj*CRT in males. The IF signals of *Sj*CRT were seen in the anterior end of oesophagus and tegument of female worms (i and ii in Fig. 4c), as well as in the ventral sucker and dorsal tegument of male worms (iii and iv in Fig. 4c).

**Fig. 4:**
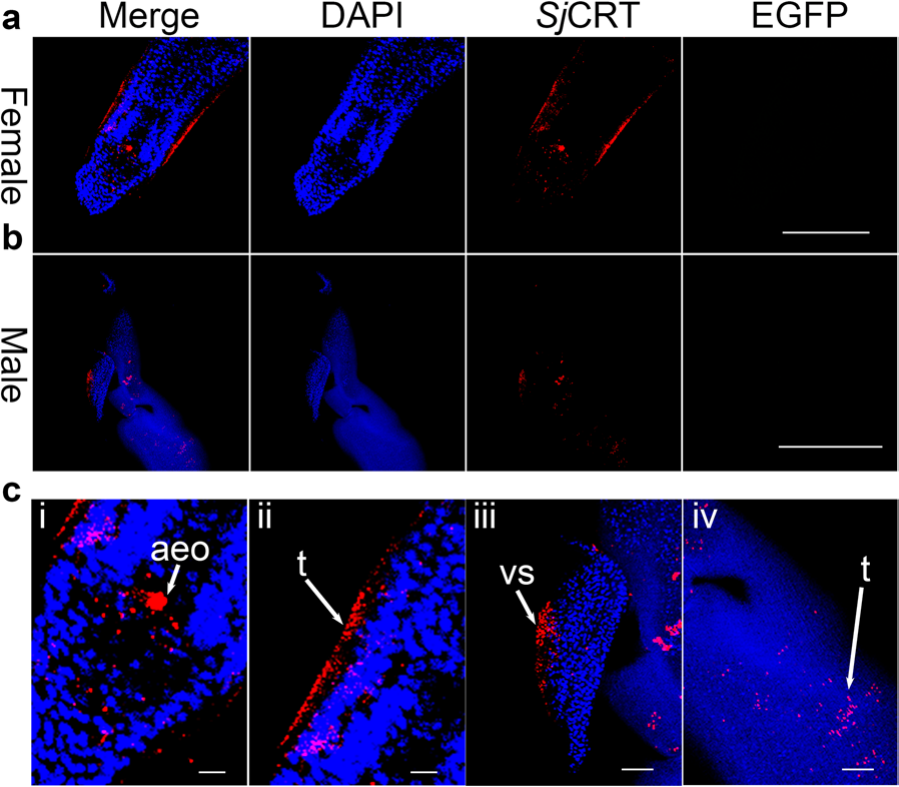
Reduction of AF for immunofluorescence assay of calreticulin (CRT) in female and male schistosomes. **a** Immunofluorescence staining for *Sj*CRT in female. Scale-bar: 100 μm. **b** Immunofluorescence staining for *Sj*CRT in male. Scale-bar: 500 μm. **c** Merged immunofluorescence images of different body regions of female and male worms; **i** the oral sucker and oesophagus of female, scale-bar: 10 μm; **ii** the tegument of female, scale-bar: 10 μm; **iii** the ventral sucker of male, scale-bar: 50 μm; **iv** the dorsal tegument of male worms, scale-bar: 50 μm. ‘Merge’ represented the merged images of DAPI/EGFP/*Sj*CRT image. DAPI column is a staining for the nuclei (blue). EGFP column is not any staining and just as a control for detecting AF. *Sj*CRT column is an immunostaining for *Sj*CRT (red). Abbreviations: aeo, the anterior end of oesophagus; t, tegument; vs, ventral sucker

Previous studies have shown that both male and female worms of *S. japonicum* can produce autofluorescence (AF) under different excitation light irradiation (405, 458, 476, 488, 514, 543 and 633 nm), with the strongest green AF (488 nm excitation) [12]. And our results (Fig. 1) shown that AF could be observed in *S. japonicum* worms at 405, 488, 561 and 640 nm excitation wavelengths, and the highest AF intensity was also excited by 488 nm wavelength. In our research, it was also shown that the AF of the tegument of male and female worms and the intestine of male worms was detectable in all channels (Fig. 1), but no AF was detected under the DAPI channel in the vitelline glands and eggs of female (Fig. 1a), which may be associated with endogenous autofluorescent substances in the vitelline glands and eggs. Common endogenous autofluorescent substances in organisms include amino acids, structural proteins, vitamins, lipopigments, flavins and porphyrins [23]. During the development of the vitelline gland of schistosome female, three types of inclusions accumulate in the vitelline cells: (1) shell globule clusters; (2) lipid droplets; (3) β-glycogen particles [24]. However, the co-localization results of AF and lipid droplet staining of vitelline cells showed that AF did not derive from lipid droplets [14].

The AF features of schistosomes have been applied in diagnosis and vitelline cells separation [13, 14], but AF is mainly an interfering signal in IFA for schistosome molecules. In the previous studies, IFAs of schistosome molecular localization showed that stronger green AF noise signal was seen in the female gonad and cecum [25], and eggshell [26, 27]; and weaker red AF was observed in female adult schistosomes [28, 29]. In our results, female and male worms had AF in four different channels (Fig. 1). All cases of AF mentioned above suggest that AF reduction is important for the IFA of schistosome molecules. In this, our assays uncovered that CuSO4, SBB, and TB had various effect (Fig. 2) for AF reduction of *S. japonicum*, and 50 mM CuSO4 reduced AF of female and 0.5% SBB reduced AF of male (Fig. 3).

CuSO_4_ and SBB are widely used chemical reagents to remove AF. In fact, the AF reduction effect both of CuSO_4_ and SBB can be seen in the samples with lipofuscin-like AF [30]. Previous studies have applied CuSO_4_ to reduce AF of schistosomes for fluorescence in situ hybridization (FISH) [19]. In our study, both CuSO_4_ and SBB were found to be effective in AF reduction for both female and male worms. However, CuSO_4_ was more effective for females while SBB was more effective for males (Fig. 2), although the mechanism of CuSO_4_ and SBB on AF reduction is not well understood. This difference of AF reduction between males and females still suggests that the reasons why females and males form AF may not be exactly the same. Triglycerides are considered a lipid associated with AF [31], and there is a significant difference in the composition of triglycerides between male and female schistosomes, with TG (52:1) being more abundant in females, while TG (58:6) is more abundant in males [32]. In addition, there was a significant difference in the uptake of the autofluorescent substance haemoglobin between male and female schistosomes [33].

The application of non-fluorescent substrate chromogenic methods (such as HRP/AP labeled secondary antibodies) for localization signal amplification of schistosome proteins can effectively avoid the interference from AF on localization signals. However, the non-fluorescence methods will also be interfered by the red blood cell products in the worm guts [34]. The localization signal of schistosome proteins were also amplified by using fluorophore labeled secondary antibody in immunofluorescence assays (IFA). However, due to deficiency of an effective AF reduction method used in the assay, the diffuse background signal presented and interfered the positive fluoresces signal recognition [35]. By increasing understanding of the phenomenon and characteristics of schistosome AF, some researchers have labelled the AF and immunofluorescence signals in schistosome IFA by experience [25]. Sometimes, experiences limited the accuracy of real positive signal judgment, especially when the immunofluorescence signal overlaps with the AF signal. In our study, through reagents screening and conditions optimization, a suitable method for AF reduction of male and female schistosomes was respectively developed. Combined with the immunofluorescence assay developed previously [15], we established an effective AF reduction-based IFA which significantly reduced the AF background signal (Additional file 3: Figure S3, Fig. 4a and b EGFP column) and highlighted the immunofluorescence signal (Fig. 4).

The immunofluorescence localization accuracy of schistosome proteins relies on reducing noise from AF and improving the specificity of the antigen–antibody reaction. In this study, through reagents screening and conditions optimization (Figs. 2, 3 and Additional file 1: Figure S1), a suitable method for AF reduction of male and female schistosomes was developed. Applied in IFA, AF could be effectively reduced (Fig. 4 and Additional file 3: Figure S3). Of course, our attention should also be paid to the specificity of antibodies when immunofluorescence localization is performed on one specific protein. In this study, the specific reaction of anti-*Sj*CRT antibody was verified using the western-blot method (Additional file 2: Figure S2). The results showed that the band was single, the weight mass conformed to the theoretical calculated value, and it was consistent with the previous study [36]. *Sj*CRT is one of immunostimulatory molecules induced Th1-polarized immune response of mice [36]. And the peptide sequences of *Sj*CRT were detected in excretory/secretory of *S. japonicum* by proteomics [37]. Therefore, *S. japonicum* in the vein of mice could regulate the polarization of Th1 cells in mice by excreting *Sj*CRT. The excretory organs of schistosomes are mainly the tegument and anterior esophageal region [38, 39]. And previous omics studies have shown that *Sj*CRT was expressed in the tegument [40]. In this study, *Sj*CRT could be observed in the tegument of male and female worms as well as in the anterior end of oesophagus of female worms by IFA (Fig. 4), our results further confirmed that *Sj*CRT is a protein produced by the excretory organs of *S. japonicum*.

Besides, in this study, the subjects were only *S. japonicum* male and female mature worms. However, AF were detected in different kinds of flukes. For example, the AF was seen in vitelline glands [14, 41] and eggs [42, 43] of *S. mansoni* and *Schistosoma haematobium*, in eggs of *Clonorchis sinensis* [44, 45] and *Opisthorchis viverrini* [46], and in vitelline glands of *Fasciola gigantica* [47, 48]. To minimize the AF, the unvisible background AF 15-day-old *C. sinensis* flukes were used for the IFA [49]. However, for samples with AF, increasing the signal-to-noise ratio allows accurate localization of the target molecules. Our method, by reducing AF noise, facilitates improved signal-to-noise ratio and perhaps can be applied to IFA in other flukes.

In summary, both female and male schistosomes have autofluorescence (AF), but the methods of AF reduction are different. Our results suggest that 50 mM CuSO_4_ reduced AF of female and 0.5% SBB reduced AF of male. The application of this method in immunofluorescence assays of schistosomes can obviously reduce AF and highlight the IF signal. Therefore, our method can improve the accuracy of functional localization for schistosome proteins and provide an idea for IFA in other flukes.

## Supplementary Information

**Additional file 1:** **Figure S1.** Autofluorescence of different channels of female and male schistosomes treated with tris-glycine (Gly) or ammonia/ethanol (AE). Scale-bar: 1000 μm.

**Additional file 2:** **Figure S2.** Western-blotting result of calreticulin (CRT) in Schistosoma japonicum. Lane 1: protein molecular mass ladder; lane 2: protein extracted from female worms; lane 3: protein extracted from male worms.

**Additional file 3:** **Figure S3.** Immunofluorescence control group of female and male worms without anti-SjCRT antibody treatment. Scale-bar: 1000 μm.

## Data Availability

All materials and data supporting these findings are contained within the manuscript and additional files 1, 2,3.

## Abbreviations

SBB: Sudan black B
TB: Trypan blue
CuSO_4_: Copper sulfate
Gly: Tris-glycine
AE: Ammonia/ethanol
TG: Triglycerides
*Sj*CRT: *Schistosoma japonicum* calreticulin
FISH: Fluorescence in situ hybridization
HRP/AP: Horseradish peroxidase or alkaline phosphatase
DAPI: 4′,6-Diamidino-2-phenylindole
Cy3: Cyanine 3
AF647: Alexa Fluor 647
AF: Autofluorescence
IF: Immunofluorescence
IFA: Immunofluorescence assay
