# Supplementary figures and images for: Reduction of autofluorescence in whole adult worms of Schistosoma japonicum for immunofluorescence assay
Source: Parasit Vectors. 2021 Oct 14;14:532. doi: 10.1186/s13071-021-05027-3 (PMC8515762; doi:10.1186/s13071-021-05027-3)

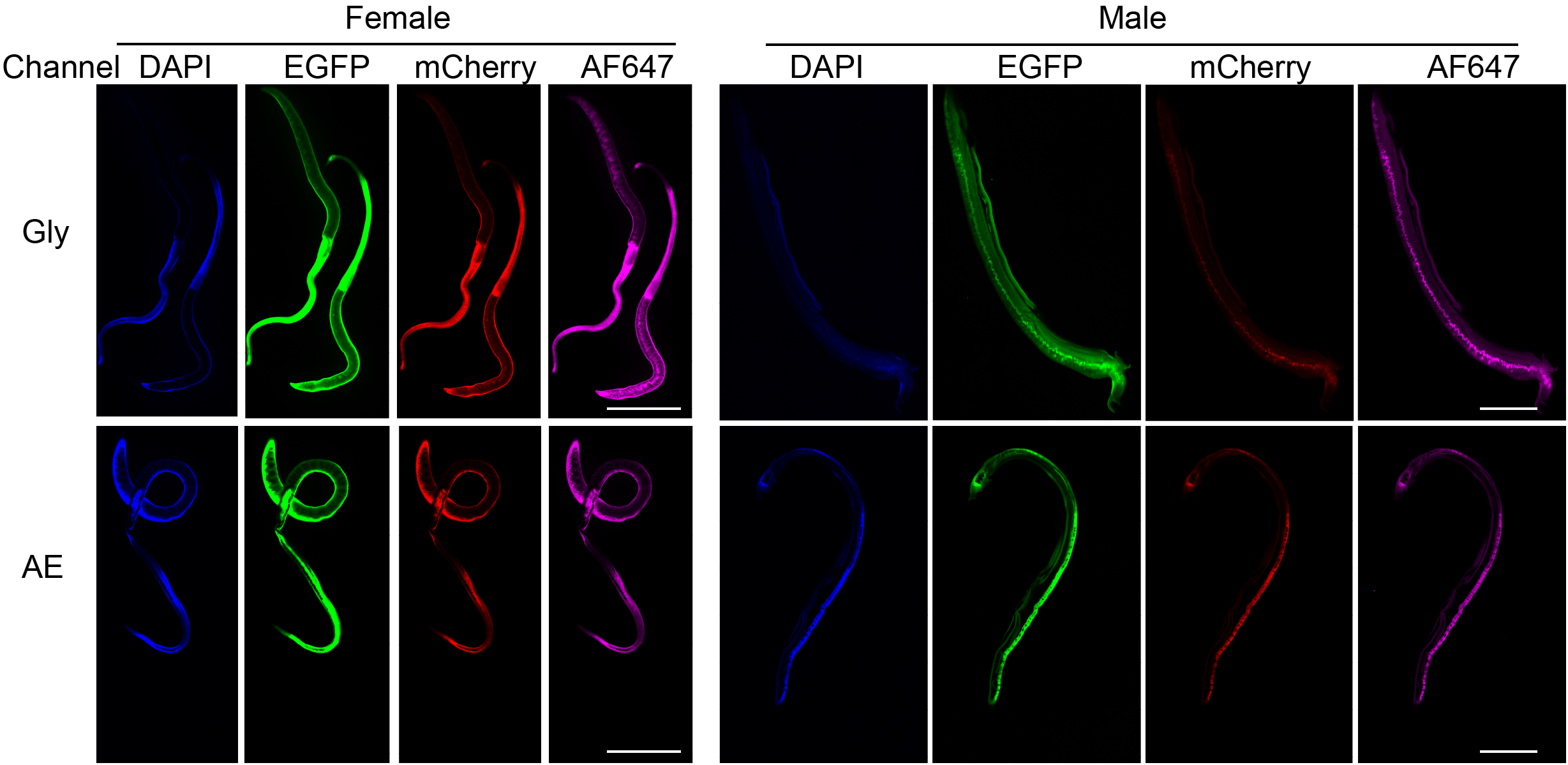

Supplement: Supplementary file 1 — Additional file 1: Figure S1. Autofluorescence of different channels of female and male schistosomes treated with tris-glycine (Gly) or ammonia/ethanol (AE). Scale-bar: 1000 μm. [file 13071_2021_5027_MOESM1_ESM.tif]

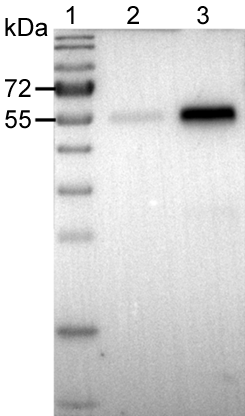

Supplement: Supplementary file 2 — Additional file 2: Figure S2. Western-blotting result of calreticulin (CRT) in Schistosoma japonicum. Lane 1: protein molecular mass ladder; lane 2: protein extracted from female worms; lane 3: protein extracted from male worms. [file 13071_2021_5027_MOESM2_ESM.tif]

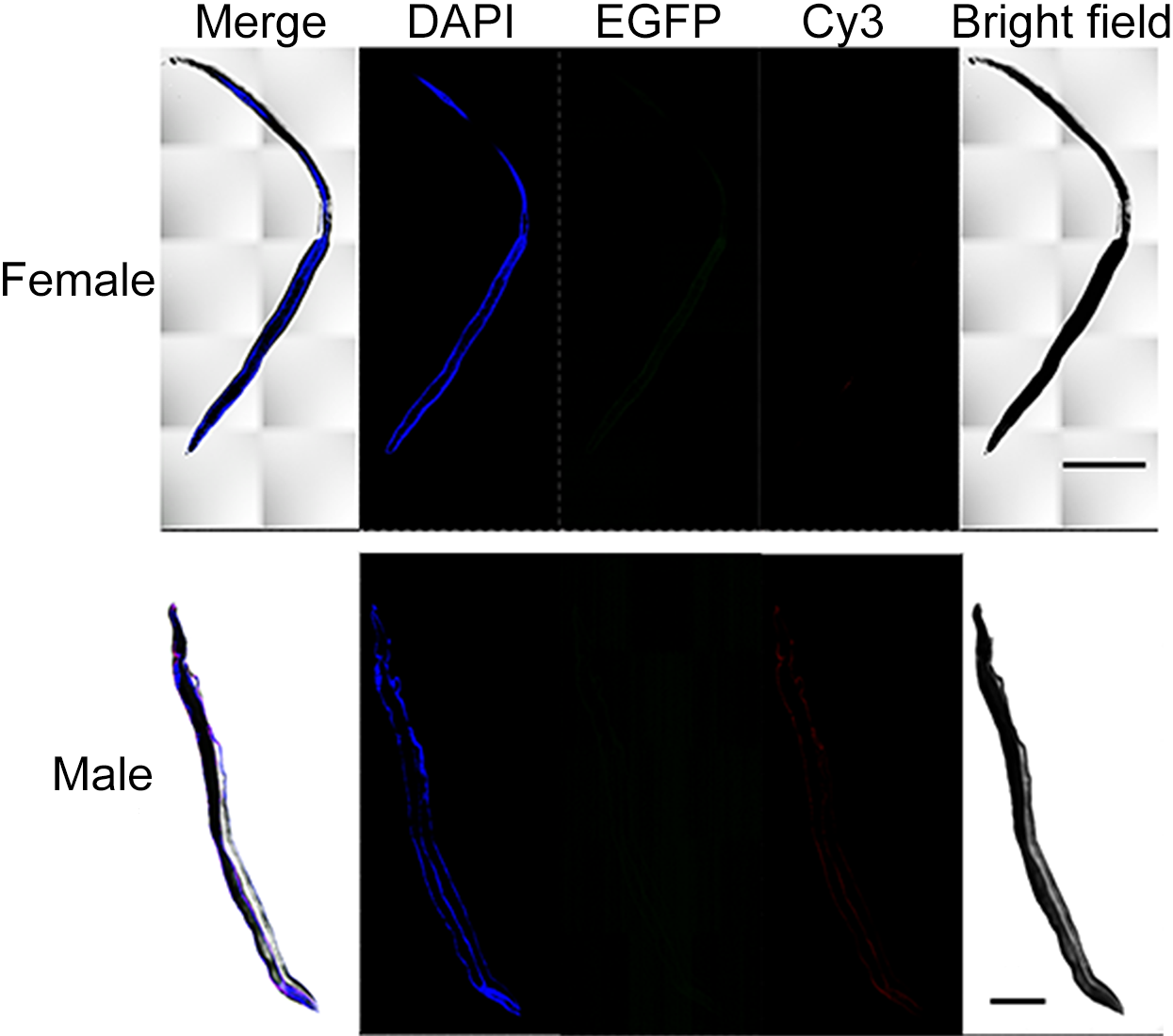

Supplement: Supplementary file 3 — Additional file 3: Figure S3. Immunofluorescence control group of female and male worms without anti-SjCRT antibody treatment. Scale-bar: 1000 μm. [file 13071_2021_5027_MOESM3_ESM.tif]
